# Molecular basis and genetics of hypohidrotic ectodermal dysplasias

**DOI:** 10.18699/VJGB-23-78

**Published:** 2023-10

**Authors:** V.A. Kovalskaia, T.B. Cherevatova, A.V. Polyakov, O.P. Ryzhkova

**Affiliations:** Research Centre for Medical Genetics, Moscow, Russia; Research Centre for Medical Genetics, Moscow, Russia; Research Centre for Medical Genetics, Moscow, Russia; Research Centre for Medical Genetics, Moscow, Russia

**Keywords:** ectodermal dysplasia, EDA, tooth agenesis, Wnt family, эктодермальная дисплазия, EDA, агенезия зубов, семейство Wnt

## Abstract

Ectodermal dysplasia (ED) is a heterogeneous group of hereditary diseases of the skin and its appendages, which are characterized by impaired development and/or homeostasis of two or more ectoderm derivatives, including: hair, teeth, nails, sweat glands and their modifications (mammary glands, for instance). The overall prevalence of ectodermal dysplasia remains precisely unknown not only in Russia, but also in the world, nor is known the contribution of individual genes to its structure. This complicates the DNA diagnosis establishment of this disease due to the lack of an accurate diagnostic algorithm and a universal cost-effective method of analysis. To date, the most highly-researched genes involved in the development of anhydrous or hypohidrotic forms of ED are EDA, EDAR, EDARADD and WNT10A. The ectodysplasin A (EDA) gene is the cause of the most common X-linked form of ED, a gene from the Wnt family (WNT10A) is responsible for the autosomal recessive form of the disease, and two other genes (EDAR and EDARADD) can cause both autosomal recessive and autosomal dominant forms. This review provides the characteristics of the genes involved in ED, their mutation spectra, the level of their expression in human tissues, as well as the interrelation of the aforementioned genes. The domain structures of the corresponding proteins are considered, as well as the molecular genetic pathways in which they are involved. Animal models for studying this disorder are also taken into consideration. Due to the cross-species genes conservation, their mutations cause the disruption of the development of ectoderm derivatives not only in humans, but also in mice, cows, dogs, and even fish. It can be exploited for a better understanding of the etiopathogenesis of ectodermal dysplasias. Moreover, this article brings up the possibility of recurrent mutations in the EDA and WNT10A genes. The review also presents data on promising approaches for intrauterine ED treatment.

## Abstract (ru)

Эктодермальные дисплазии – гетерогенная группа наследственных заболеваний кожи и ее придатков, которые характеризуются нарушением развития и/или гомеостаза двух и более производных эктодермы, включая: волосы, зубы, ногти, потовые железы и их модификации (например, молочные железы). Общая распространенность эктодермальных дисплазий остается точно неизвестной не только в России, но и в мире, так же как и вклад отдельных генов в ее структуру. Это затрудняет ДНК-диагностику данного заболевания ввиду отсутствия строгого алгоритма диагностики и универсального, экономически выгодного метода анализа. На сегодняшний день наиболее изученными генами, вовлеченными в развитие ангидротической или гипогидротической форм эктодермальной дисплазии являются EDA, EDAR, EDARADD и WNT10A. Ген эктодисплазина А (EDA) служит причиной самой частой Х-сцепленной формы эктодермальной дисплазии, ген из семейства Wnt (WNT10A) отвечает за аутосомно-рецессивную форму заболевания, а два других гена (EDAR и EDARADD) могут быть причиной как аутосомно-рецессивных, так и аутосомно-доминантных форм. В настоящем литературном обзоре приведены характеристика генов, вовлеченных в эктодермальную дисплазию, спектры их мутаций, уровень их экспрессии в тканях человека, а также взаимосвязь вышеупомянутых генов друг с другом. Обсуждается также доменная структура соответствующих белков, рассмотрены молекулярно-генетические пути, в которые они преимущественно вовлечены, и описаны животные модели для изучения данной патологии. Ввиду межвидовой консервативности упомянутых генов, мутации в них вызывают нарушения развития производных эктодермы не только у человека, но и у мышей, коров, собак и даже рыб, что может быть использовано для лучшего понимания этиопатогенеза эктодермальных дисплазий. Более того, в статье поднимаются вопросы о возможных частых мутациях в генах EDA и WNT10A. Приведены также данные касаемо разрабатываемых перспективных подходов к внутриутробному лечению эктодермальной дисплазии.

## Introduction

Ectodermal dysplasia (ED) refers to a diverse set of molecular genetic disorders that share a common feature of developmental abnormalities or imbalances affecting two or more ectodermal structures (Wright et al., 2019). Despite the fact that the ectoderm determines the development of many organs and tissues, such as: the central and peripheral nervous system, pituitary gland, olfactory neuroepithelium, melanocytes, tooth enamel, epidermis, including sweat glands, hair, nails, ED primarily affects the latter group of ectodermal derivatives.

The exact prevalence of ED is uncertain due to limited research and differing classification criteria across countries. Nonetheless, some estimate that it may affect as many as 70 out of 100,000 newborns (Itin, Fistarol, 2004). Reported data for the Danish population, collected from 1995 to 2010, indicate that the prevalence of ectodermal dysplasia corresponds to 21.9 per 100,000, and molecularly confirmed X-linked ectodermal dysplasia – 1.6 per 100,000 (Nguyen-Nielsen et al., 2013). Thus, ectodermal dysplasias, although they are not among the most common hereditary diseases, have a widespread presence and make a significant contribution to the structure of dental, dermatological, and genetic pathologies.

This group of pathological conditions may have been known since the end of the 18th century, however, the first documented case of ectodermal dysplasia dates back to 1838, when Wedderburn, in a letter to Charles Darwin, described 10 men from an Indian family suffering from partial absence of teeth, baldness and excessive dry skin (Felsher, 1944). In 1848, two additional patients were reported by Thurman, and another case was documented by Guilford in 1883. However, only in 1929 the term “hereditary ectodermal dysplasia” was introduced by Weech, who also introduced the term “anhidrotic” to describe individuals with ectodermal dysplasia who exhibit a diminished ability to sweat. This term was subsequently replaced by “hypohidrotic” (Weech, 1929). Following an analysis of 19 affected families in 1937, Siemens concluded that the genetic etiology of ectodermal dysplasia could not be explained by a single gene and one mode of inheritance. Thus, it was realized that both dominant and recessive forms of the disease were present, along with sex-linked forms that exhibited phenotypic overlap but did not entirely replicate it (Siemens, 1937). In 1939, Clouston noticed that despite similar clinical data patients with manifestations of ectodermal dysplasia may differ significantly from each other in the extent of sweat gland development. As a result, he classified them into two broad categories: hypohidrotic type, which was limited to only 4 cases, and hidrotic ectodermal dysplasia, which encompassed over 50 patients (Clouston, 1939).

Further study of this nosological unit resulted in the development of the initial clinical classification by Freire-Maia and Pinheiro (Freire-Maia, 1971; Freire-Maia, Pinheiro, 1988), which has been widely employed in routine medical practice. Their classification was based on the principle of involvement of certain ectodermal structures in the pathological process. They assigned to group A all conditions in which at least two classical derivatives of the ectoderm were affected, such as: hair, teeth, nails and sweat glands. Diseases assigned to group B included deviations in only one of the four structures mentioned above and one additional ectodermal defect, such as abnormalities of the ears, lips, or palmar and plantar hyperkeratosis. The condition, which was characterized by the presence of only ectodermal signs, they called pure ectodermal dysplasia. The combination of ectodermal signs with other anomalies was called ectodermal dysplasia syndrome by the authors. In addition, all the classical structures of the ectoderm were numbered, where 1 – hair, 2 – teeth, 3 – nails, 4 – sweat glands, in order to distinguish further the main groups of ED: ED1 – trichodysplasia, ED2 – dental dysplasia, ED3 – onychodysplasia, ED4 – dyshidrosis (Deshmukh, Prashanth, 2012). It is worth mentioning that the classification proposed by Freire-Maia and Pinheiro did not consider the molecular and genetic aspects of ectodermal dysplasia and necessitated revision with the emergence of next-generation sequencing techniques and advancements in genomic medicine.

At the end of 2019, a group of international experts associated with the National Foundation for Ectodermal Dysplasias (NFED) published a revised classification of ectodermal dysplasia (ED) in the American Journal of Medical Genetics. This updated classification system is based on the molecular pathways involved in the development of ED and provides a more precise list of pathologies than previous classifications. Specifically, the new classification identifies 102 syndromes that fall under the definition of ectodermal dysplasia, reflecting a more comprehensive understanding of this complex condition (Wright et al., 2019). In addition to non-syndromic ectodermal dysplasia, it included such heterogeneous syndromes as: Coffin–Siris, Dubowitz, Hallermann– Streiff, Gorlin–Goltz, Johanson–Blizzard and others, which does not meet the criteria set by domestic terminology. In the Russian Federation, the term “ectodermal dysplasia” typically refers only to non-syndromic forms of the condition, specifically anhidrotic (hypohidrotic) and hidrotic forms (Kozlova, Demikova, 2007). However, it is worth noting that these isolated forms are fully consistent with the molecular etiology proposed by experts from NFED.

**Fig. 1.**
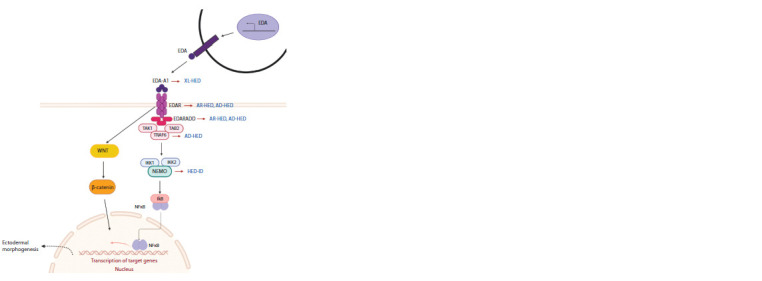
The major proteins involved in the development of ectodermal structures EDA is expressed on the cell surface, but its extracellular domain can be proteolytically cleaved to form a soluble signaling molecule that binds to the ectodysplasin receptor (EDAR). EDAR interacts with the EDARADD protein, and further downstream signaling via activation of the NFκB pathway leads to the expression of genes specific to the epidermis, hair, teeth, and nails. XL-HED – X-linked hypohidrotic ectodermal dysplasia, AD-HED – autosomal dominant hypohidrotic ectodermal dysplasia, AR-HED – autosomal recessive hypohidrotic ectodermal dysplasia, HED-ID – hypohidrotic ectodermal dysplasia with immunodeficiency.

The current understanding of ED suggests the involvement of four major signaling pathways: EDA-mediated, WNT-, NF-κB-, and TP63-mediated pathways. Of these, only the first three have been implicated in the development of anhidrotic forms of ectodermal dysplasia (Fig. 1) (Mikkola, 2009; Sadier et al., 2015; Wright et al., 2019).

## Major genes involved in the development of ectodermal dysplasia

The human EDA gene (also known as ED1, HED, EDA1, EDA2, HED1, ODT1, XHED, ECTD1) is a protein-coding gene responsible for the synthesis of ectodysplasin A, a type II transmembrane protein belonging to the tumor necrosis factor (TNF) family, which is involved in the transmission of epithelial-mesenchymal signals during the morphogenesis of ectodermal structures in humans (Bayés et al., 1998; Mikkola, Thesleff, 2003).

The EDA (ectodysplasin A) gene is mapped on the X chromosome, at the Xq13.1 locus, according to the main transcript (NM_001399.5), it contains 8 exons, with start and stop codons in the first and last exons, respectively. A total of 8 protein-coding isoforms have been described, differing in length and function, but isoform 1 (EDA-A1), consisting of 391 amino acids, is the main one and is the ligand for the EDAR receptor. Another isoform, known as EDA-2, is distinguished by the absence of Val307 and Glu308 in the TNF domain and binds only to EDA2R, ensuring the subsequent correct postembryonic functioning of various structures and tissues (Kere et al., 1996). Both isoforms, EDA1 and EDA2, through the EDAR and EDA2R receptors activate the NFκB signaling pathway, but only the EDA1/EDAR interaction is important in the development of ectoderm derivatives and the disease (Newton et al., 2004). The precise reason why the disruption of EDA-A2/XEDAR interaction does not lead to the ectodermal dysplasia phenotype is currently unknown and requires further investigation, however, studies have shown that EDA-A2 is expressed primarily in aging adipose tissue, arteries, heart, lungs, muscle, and skin, and may also regulate glucose metabolism, and serves as a predictor of steatosis aggravation in patients with non-alcoholic fatty liver disease (Yang et al., 2015; Cai et al., 2021).

**Fig. 2.**
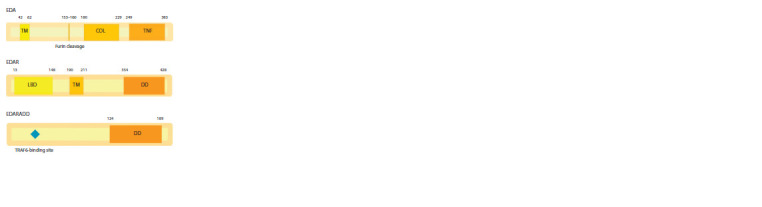
Domain structure of the main proteins involved in the EDA-mediated pathway. TM – transmembrane domain; Furin cleavage – Furin cleavage site; COL – collagen domain; TNF – tumor necrosis factor domain; LBD – ligand-binding domain; DD – death domain.

Besides the C-terminal TNF domain (249–383 aa), ectodysplasin A contains a collagen domain (180–229 aa), a furin cleavage site (153–160 aa), and a transmembrane N-terminal domain (42–62 aa) (Chen et al., 2001; www.ncbi.nlm.nih.gov/ Structure/cdd/wrpsb.cgi; www.uniprot.org/uniprot/Q92838) (Fig. 2). Ectodysplasin A, as a member of the TNF-ligand family, can function locally through direct intercellular contacts as a complete membrane form. However, it predominantly acts in its secreted form, which is generated through proteolytic cleavage at a furin consensus site that releases the C-terminal part of the protein as a soluble trimeric ligand. This ligand then initiates downstream signaling by activating various proteins (Elomaa, 2001) (see Fig. 1).

EDAR is another key protein in this molecular genetic pathway, encoded by the gene of the same name at the chr2q12.3 locus. As for the topology, the ectodysplasin A receptor has an extracellular part, including a ligand-binding domain (LBD) (13–148 aa), encoded by exons 2–5 and a cytoplasmic part, represented by a death domain, encoded by exon 12 (354–428 aa) for interaction with the β-isoform of EDARADD (Sadier et al., 2015; Zhang et al., 2020; www. ncbi.nlm.nih.gov/Structure/cdd/cdd.shtml). The latter, in turn, through the EDAR-associated death domain (124–189 aa) and the TRAF6-binding site (27–31 aa) leads to subsequent activation of the NF-κB pathway (Morlon et al., 2005; Asano et al., 2021) (see Fig. 1).

The high degree of similarity between the human and mouse genes facilitated the generation of several mutant mouse lines in the 1990s (Headon, Overbeek, 1999; Trzeciak, Koczorowski, 2015). It was observed that subjects lacking EDA2R(XEDAR) did not exhibit any symptoms of ectodermal dysplasia (Newton et al., 2004). However, individuals harboring recessive (downless) and dominant (Sleek) mutations in the EDAR gene demonstrated significant disruption in the development of ectodermal structures, such as sparse hair, absence of cover behind the ear, and presence of abnormal teeth, particularly incisors (Crocker, Cattanach, 1979). In the phenotype of Tabby mutant (analog of human EDA) male mice, there were found alopecia areata behind the ears, tail alopecia, an absence of some vibrissae, abnormal fur texture due to the absence of zigzag-shaped and protective hairs, and an absence of sweat glands normally found on the paw pads (Ferguson et al., 1997; Srivastava et al., 1997). In crinkled and swh/swh mice, due to homozygous variants in the EDARADD gene, a similar ectodermal dysplasia phenotype was observed, and females were unable to feed offspring due to underdevelopment of the mammary glands (Yan et al., 2002; Kuramoto et al., 2005, 2011).

The highly conserved nature of the EDA-mediated pathway has enabled the identification of a common phenotype in other vertebrates, as well as distinct mutations in genes of interest (Pantalacci et al., 2008). Notably, a long deletion spanning exon 3 of the EDA gene was identified in four male calves, resulting in a significant reduction in hair density on the head, auricles, neck, back, and tail. These areas of the body exhibited sparse hair growth, while teeth abnormalities such as partial adentia and conical teeth were also observed (Drögemüller et al., 2001, 2002). In male dogs with a hemizygous mutation in the splicing acceptor site of exon 8 of the EDA gene, all sweat glands were absent, they were completely devoid of hair in the frontal part and in the pelvic region on the back. Most premolars and some incisors were missing, and the present teeth were mostly conical. Moreover, affected dogs showed increased morbidity and mortality from pulmonary infectious diseases compared to other dogs in the same environment (Casal et al., 2005). Zebrafish and medaka mutants with disturbances in EDA signaling (EDA and EDAR genes) were observed to have lost fins and scales, lacked teeth, or had abnormally-shaped teeth (Harris et al., 2008; Atukorala et al., 2010). The situation was similar with marine and freshwater sticklebacks: marine representatives of Gasterosteus aculeatus, in which the expression of the EDA gene is much higher, demonstrated a more developed cover with 32 lateral plates, while freshwater individuals were limited to 0–9 lateral plates (O’Brown et al., 2015). Based on these data, the EDA pathway probably controls the development of ectoderm derivatives in all vertebrates (Sadier et al., 2014).

EDA is mostly expressed in endocrine organs (adrenals, thyroid, ovaries), various parts of the brain and heart, the lowest level of expression is observed in blood cells (TPM 0.18). For cultured fibroblasts, this indicator is 0.89, which makes them the most accessible object for studying EDA-transcripts (www.gtexportal.org). Expression of EDAR and EDARADD predominantly occurs in the bladder, esophageal mucosa, and skin. However, it is also more efficient to study EDARADD expression patterns on a culture of fibroblasts, while studying the structure of mRNA and splicing disorders of EDAR-transcripts is possible mainly only when using blood leukocytes (www.gtexportal.org).

According to the HGMD database, mutations in EDA, EDAR and EDARADD are relatively evenly distributed throughout the genes and affect all significant domains (www.hgmd.cf. ac.uk). To date, 371 pathogenic variants have been described in the EDA gene, 83 in EDAR, and 19 in EDARADD, including missense and nonsense mutations that make up the major part, deletions and insertions, including gross ones, as well as splicing variants, affecting both canonical splicing sites and leading to activation of cryptic ones (www.hgmd.cf.ac.uk).

To date, there is no record of classical recurrent mutations in the genes related to the EDA-mediated pathway in any population. However, some researchers have reported that variants in EDA affecting amino acids R155 and R156 at overlapping furin cleavage sites may amount to from 7 to 30 % (Vincent et al., 2001; Chaudhary et al., 2022). This phenomenon can, in particular, be explained by the presence of a CpG rich region in exon 3 in arginine codons 155, 156, which, when methylated, causes the so-called C-T transition (Chen et al., 2001).

Another notable observation is that the majority of mutations occurring in the EDA gene lead to X-linked hypohidrotic ectodermal dysplasia (OMIM 305100), characterized by classic symptoms such as scalp hypotrichosis, nail dystrophy, oligodontia with conical incisors, and hypohidrosis (www.omim. org). While male patients exhibit a more severe phenotype, clinical manifestations can also be observed in females, even in the absence of an unequal pattern of X-chromosome inactivation (Vincent et al., 2001). Up to 70 % of heterozygous female carriers of pathogenic variants in the EDA gene demonstrate one or more disorders: some degree of hypotrichosis, reduced sweating, missing one or more teeth, underdevelopment of the mammary glands, or problems with breastfeeding – the latter, however, can only be fully assessed after puberty or pregnancy, respectively (Wahlbuhl-Becker et al., 2017; Wohlfart et al., 2020). Moreover, even within the same family, there is a certain variability in the phenotype (Cañueto et al., 2011; Han et al., 2020). Cases of selective tooth agenesis (OMIM 313500) of the X-linked mode of inheritance, which were also caused by pathogenic variants in the EDA gene, are described. Despite the fact that mutations leading to this phenotype have been described in different protein domains of ectodysplasin A, the presence of residual activity of the protein and the possibility of its binding to the EDAR receptor is probably the key factor (Mues et al., 2010).

For the EDAR and EDARADD genes, both autosomal dominant and autosomal recessive forms of anhidrotic ectodermal dysplasia have been described. Some authors believe that dominant mutations are mainly localized in the domains of protein-protein interactions, which leads to disruption of oligomerization and a dominant negative effect (Sadier et al., 2014). However, this assumption is not fully justified. Thus, functional analysis of missense mutations p.D120Y, p.L122R, and p.D123N located near the EDARADD death domain proved not only their dominant nature, but also the ability to significantly reduce the interaction with TRAF6 and suppress the subsequent activation of NF-κB. The p.E152K mutation in the heterozygous state, located directly in the EDAR-associated death domain, on the contrary, was recessive and showed only a slight decrease in affinity for TRAF6 (Asano et al., 2021).

The V370A variant is a conservative amino acid substitution in the EDAR gene identified in Asian and Latin American populations by whole genome sequencing (Park et al., 2012). It is believed to be a gain-of-function mutation that leads to a 2-fold increase in the activation of the NF-κB pathway (Kataoka et al., 2021) and, accordingly, correlates with increased hair thickness and special tooth morphology in representatives of Asia and indigenous peoples of the USA (Bryk et al., 2008). An interesting fact is that this variant was selected, presumably in Central China, about 30,000 years ago, and the presence in the genotype of pathogenic variants in the EDA gene in the presence of V370A reduces the severity of clinical manifestations of anhidrotic ectodermal dysplasia (Cluzeau et al., 2011).

NEMO is another protein involved in the pathogenetic cascade. Due to the fact that NF-κB also controls the immune response and apoptosis, the clinical manifestations of mutations in the NEMO gene are not only limited to damage to ectodermal structures, but also include immune system disorders, with the development, in particular, of anhidrotic ectodermal dysplasia with immunodeficiency 1 (OMIM 300291) (Smahi et al., 2002).

Mutations in the WNT10A gene are the most common cause of non-syndromic selective tooth agenesis (Xu et al., 2017; Yu et al., 2019), but are also associated with the development of hypohidrotic ectodermal dysplasia, odonto-onychodermal dysplasia, and Schöpf–Schulz–Passarge syndrome. The WNT10A gene encodes a protein of the same name, a component of the canonical Wnt/β-catenin signaling pathway that plays an important role in several stages of dental morphogenesis, including activation of the mesenchymal odontogenic potential during early tooth development, as well as the induction and maintenance of primary and secondary enamel nodes (Xu et al., 2017). Conversations are currently in progress regarding the contribution of Wnt signaling to the development and formation of hair follicles and skin structures (Adaimy et al., 2007). The human Wnt-family includes genes that show significant similarity to mouse wingless genes, and therefore alopecia is consistently observed in WNT10Adeficient mice (WNT10A–/–), besides growth retardation, kyphosis, and reproductive dysfunction (Wang et al., 2018).

Currently, 94 variants for WNT10A are described in the HGMD database, however, only p.Cys107Ter (rs121908119) and p.Phe228Ile (rs121908120) variants have been suggested to be located in hotspots. These mutations were the most common among patients of Polish and Italian origin with WNT10A-mediated ectodermal dysplasia (Castori et al., 2010; Mostowska et al., 2012).

## Treatment approaches of ectodermal dysplasia

At present, treatment options for ED are primarily aimed at managing the symptoms to prevent complications. A targeted and effective treatment approach is yet to be developed. However, a phase 2 clinical trial (NCT04980638) involving the intra-amniotic administration of ER004 to male fetuses with confirmed X-linked ectodermal dysplasia is currently underway. ER004 is a novel signaling protein replacement molecule that has been specifically designed to bind with high affinity to the endogenous EDAR receptor. ER004 is believed to function by providing a replacement for the deficient ectodysplasin A protein in patients who have pathogenic variants in the EDA gene. This replacement aims to facilitate the normal development of essential ectodermal structures. The proposed administration method is intra-amniotic, with a suggested dose of 100 mg/kg fetal weight per injection. Treatment would consist of a total of three injections administered at intervals of three weeks, beginning at 26 weeks of gestation. In order to evaluate the long-term efficacy and safety of the treatment, individuals will be monitored for a period of 5 years. The end of testing is scheduled for April 2029 (www.clinicaltrials.gov/ ct2/show/NCT04980638).

EDI200 is an additional drug currently being developed. It is expected to be administered postnatally, between days 2 and 14 of life, in male patients diagnosed with X-linked ectodermal dysplasia. The treatment would consist of five injections, each containing 3 mg/kg of the human ectodysplasin A molecule. Similar to ER004, EDI200 also targets the activation of the EDA-mediated pathway. In vivo experiments conducted on XLHED-affected animals have demonstrated that a course of EDI200 therapy, whether administered prenatally or postnatally, can correct EDA deficiency. To evaluate long-term efficacy and safety, individuals treated with EDI200 will be monitored until they reach the age of 10 years (until March of 2025) (www.clinicaltrials.gov/ct2/show/NCT01992289).

## Conclusion

In Russia, molecular genetic studies of ectodermal dysplasia have not yet been carried out; the contribution of mutations of various ED genes remains unknown. The study of the full spectrum of mutations in the ED genes will allow developing an algorithm for the molecular genetic diagnosis of ectodermal dysplasia.

## Conflict of interest

The authors declare no conflict of interest.
